# Polycystic Ovary Syndrome: A Comprehensive Exploration of Diagnosis Experience in Saudi Women

**DOI:** 10.3390/jcm13175305

**Published:** 2024-09-07

**Authors:** Norah Alqntash, Alya AlZabin, Ebtesam Almajed, Kayan Alotaibi, Ghada Alhindi, Sayed Ibrahim Ali, Hanadi Bakhsh

**Affiliations:** 1 College of Medicine, Princess Nourah bint Abdulrahman University, Riyadh 11671, Saudi Arabia; nhalqntash@gmail.com (N.A.); alzabinalya@gmail.com (A.A.); 8ebtesamalmajed@gmail.com (E.A.); k.y.alotaibi7@gmail.com (K.A.); 2 Department of Obstetrics and Gynecology, Ibn Sina National College for Medical Studies, Jeddah 22421, Saudi Arabia; alhindi.ghada19@gmail.com; 3 College of Medicine, King Faisal University, Hofuf 31982, Saudi Arabia; seali@kfu.edu.sa; 4 Obstetrics and Gynecology Department, College of Medicine, Princess Nourah bint Abdulrahman University, Riyadh 11564, Saudi Arabia

**Keywords:** polycystic ovary syndrome, diagnostic experience, patient satisfaction, Saudi Arabia, women’s health

## Abstract

**Background/Objectives:** Polycystic Ovary Syndrome (PCOS) is a prevalent endocrine disorder among women of reproductive age, characterized by symptoms such as menstrual irregularities, hyperandrogenism, and polycystic ovaries. This study aimed to explore the diagnostic experiences of women with PCOS in Saudi Arabia, evaluating the timeline to diagnosis, the adequacy of information provided, and overall patient satisfaction with the healthcare process. **Methods:** A cross-sectional online survey was conducted with 1182 women diagnosed with PCOS across Saudi Arabia. The survey collected data on sociodemographic characteristics, the timeline from symptom onset to diagnosis, the number of healthcare visits required for diagnosis, and satisfaction with the information and support provided during the diagnostic process. Statistical analyses, including linear regression, were performed to identify factors influencing patient satisfaction. **Results:** The study found that 43.2% of participants sought medical attention within a year of symptom onset, yet significant delays in diagnosis were common, with 28.6% of women waiting six months or more after seeking medical care. Only 42.7% of women reported receiving adequate information at diagnosis, and satisfaction levels varied across different aspects of care. Key predictors of lower satisfaction included marital status and longer time since diagnosis, while quicker diagnosis and more healthcare visits before diagnosis positively influenced satisfaction. **Conclusions:** The findings highlight critical gaps in the diagnostic process and patient education for PCOS in Saudi Arabia. The widespread dissatisfaction with the information provided underscores the need for improved patient-centered care, comprehensive education, and standardized diagnostic protocols. Addressing these issues could enhance patient satisfaction and lead to better management of PCOS, both in Saudi Arabia and globally.

## 1. Introduction

Polycystic Ovary Syndrome (PCOS) is a prevalent endocrine disorder that affects women of reproductive age. It is characterized by a combination of symptoms including menstrual irregularities, hyperandrogenism, and the presence of polycystic ovaries [1]. Menstrual irregularities can manifest as infrequent, prolonged, or absent menstrual cycles [2]. Hyperandrogenism is evidenced by elevated levels of hormones, leading to symptoms such as hirsutism, acne, and alopecia [3]. Polycystic ovaries are typically identified through ultrasound imaging, showing multiple small cysts in the ovaries. Despite its name, the presence of ovarian cysts is not necessary for a PCOS diagnosis [4].

Hyperandrogenism is a hallmark feature of PCOS and is defined by elevated levels of androgens, such as testosterone, in the bloodstream. Clinically, hyperandrogenism manifests as hirsutism, which is excessive hair growth on the face, chest, and back; acne; and alopecia, or hair thinning [3]. These symptoms are not only physically distressing but also contribute to the psychological burden experienced by many women with PCOS.

In addition to hyperandrogenism, menstrual irregularities are another primary symptom, often presenting as oligomenorrhea (infrequent periods), amenorrhea (absence of periods), or prolonged menstrual cycles [5]. The presence of polycystic ovaries, typically identified via ultrasound, further supports the diagnosis but is not mandatory for it. These varied presentations underscore the complexity of PCOS and the challenges it poses for timely diagnosis and management [6].

Globally, PCOS affects approximately 5–10% of women of reproductive age, making it one of the most common endocrine disorders in this demographic [7]. The prevalence of PCOS can vary depending on the population studied and the diagnostic criteria used. For instance, some studies report higher prevalence rates in certain ethnic groups, while others find more consistent rates across diverse populations [8]. PCOS commonly manifests during adolescence, although it can remain undiagnosed for several years. Women in their twenties and thirties are often diagnosed when they seek medical advice for irregular menstrual cycles, infertility, or other related symptoms [9,10].

PCOS has broad health implications that extend beyond reproductive health. Women with PCOS are at an increased risk for several metabolic disorders, including insulin resistance, type 2 diabetes, and obesity [11,12]. These metabolic issues can contribute to a higher likelihood of developing cardiovascular diseases, such as hypertension and dyslipidemia [13]. Additionally, the hormonal imbalances associated with PCOS can lead to long-term reproductive issues, including infertility and complications during pregnancy. Psychologically, the condition is often associated with anxiety, depression, and body image concerns [14].

The impact of PCOS on the quality of life and daily functioning of affected women is significant. The chronic and often visible symptoms, such as hirsutism and acne, can lead to decreased self-esteem and social withdrawal [13,15]. The metabolic and reproductive complications add further stress, contributing to mental health challenges. Women with PCOS often report a diminished quality of life due to the multifaceted nature of the condition, which affects their physical, emotional, and social well-being [16].

The diagnosis of PCOS typically relies on established criteria, with the Rotterdam criteria being the most widely used. According to the Rotterdam criteria, a diagnosis of PCOS is made if at least two of the following three criteria are met: oligo/anovulation, hyperandrogenism, and polycystic ovaries [17]. Oligo/anovulation refers to infrequent or absent ovulation, leading to irregular menstrual cycles [18]. Hyperandrogenism can be clinically assessed through physical signs such as hirsutism or biochemically through elevated androgen levels [19]. Polycystic ovaries are identified via ultrasound, showing multiple small follicles [20].

Despite these criteria, diagnosing PCOS can be challenging due to the variability in its symptoms and their overlap with other conditions. Symptoms can vary widely in severity and may change over time, making it difficult for healthcare providers to recognize the syndrome early [21]. Conditions such as thyroid disorders, hyperprolactinemia, and congenital adrenal hyperplasia can present with similar symptoms, complicating the diagnostic process [22]. Additionally, the subjective nature of some symptoms, like hirsutism, can lead to inconsistent clinical evaluations. As a result, many women experience delays in receiving an accurate diagnosis [23].

Misdiagnosis and diagnostic delays are common issues faced by women with PCOS. These challenges are often exacerbated by a lack of awareness and understanding of PCOS among healthcare providers [24,25]. Women frequently report being misdiagnosed with other conditions before receiving a correct PCOS diagnosis, leading to prolonged periods of untreated symptoms [26]. The variability in symptom presentation further complicates the diagnostic process, contributing to these delays. Misdiagnosis can also result in inappropriate treatments, which may not address the underlying hormonal and metabolic issues associated with PCOS [24].

Improving the diagnostic process for PCOS is essential for providing timely and effective care. Increased awareness and education about PCOS among healthcare providers can help reduce the incidence of misdiagnosis and delays [27]. Additionally, refining diagnostic criteria to better capture the heterogeneity of the condition could lead to more accurate diagnoses [28]. By addressing these diagnostic challenges, healthcare providers can ensure that women with PCOS receive the appropriate treatments and support they need to manage their symptoms and improve their quality of life [29].

### 1.1. Aim of the Study

The aim of this study is to explore the diagnostic experiences of women diagnosed with Polycystic Ovary Syndrome (PCOS) in Saudi Arabia. The study seeks to understand the timeline and process leading to a PCOS diagnosis, evaluate the adequacy of information provided to patients, identify factors influencing patient satisfaction, and investigate regional variations in diagnostic experiences and satisfaction levels. Ultimately, the goal is to identify areas for improvement in the diagnostic process and patient care to enhance the overall experience and outcomes for women with PCOS.

### 1.2. Research Questions

1. What is the typical duration between the onset of symptoms and receiving a PCOS diagnosis among women in Saudi Arabia?
2. How adequate is the information provided to women at the time of their PCOS diagnosis, and what are the common information gaps?
3. What factors influence the satisfaction levels of women with their PCOS diagnosis experience?

## 2. Materials and Methods

### 2.1. Study Design

This study was designed as an online-based cross-sectional survey to explore the diagnosis experiences of women diagnosed with Polycystic Ovary Syndrome (PCOS) in Saudi Arabia. The cross-sectional design was chosen to capture a snapshot of the current experiences and perceptions of women at various stages post-diagnosis, providing valuable insights into the factors influencing their satisfaction and the challenges they face.

### 2.2. Sample and Sampling Technique

#### 2.2.1. Sample

The study targeted women aged 18 years and older who had been diagnosed with Polycystic Ovary Syndrome (PCOS) by a gynecologist and were residing in Saudi Arabia at the time of the survey. The inclusion criteria ensured that the participants had a confirmed diagnosis of PCOS and could provide relevant insights into their diagnostic experiences. Women who did not meet these criteria were excluded from the study.

The final sample size consisted of 1182 women. This sample size was determined to be sufficient based on the estimated prevalence of PCOS in Saudi Arabia and the power calculations performed using the Epi Info™ tool (version 7.2) provided by the Centers for Disease Control and Prevention (CDC). With a power of 80%, a confidence level of 95%, and an estimated PCOS prevalence of 53.7%, the minimum required sample size was calculated to be 382 participants. To ensure robustness and generalizability of the findings, a larger sample size was targeted and achieved.

#### 2.2.2. Sampling Technique

A convenience sampling technique was employed to recruit participants for the study. This method was chosen due to its practicality and efficiency in reaching a large number of respondents within a short period. Given the online nature of the survey, convenience sampling allowed for broad national coverage, ensuring participation from various regions across Saudi Arabia, including central, western, eastern, northern, and southern areas.

Participants were recruited through various channels, including social media platforms, online PCOS support groups, and networks within medical institutions. The survey link was distributed widely to maximize reach and ensure diversity in the sample. Additionally, participants were encouraged to share the survey link with other women diagnosed with PCOS, further enhancing the study’s reach and representation.

### 2.3. Data Collection Tool

#### 2.3.1. Development of the Questionnaire

The primary data collection tool for this study was a structured, self-administered online questionnaire. The questionnaire was developed based on a comprehensive review of the existing literature on PCOS diagnosis and patient satisfaction. It was adapted from a validated questionnaire previously used in similar studies to ensure relevance and reliability. The questionnaire was designed to capture detailed information on the sociodemographic characteristics of the participants, their experiences with the PCOS diagnostic process, and their satisfaction with the information and support they received.

#### 2.3.2. Structure of the Questionnaire

The questionnaire was divided into two main sections:

1. Sociodemographic Data:
  ○ This section included questions on age, age at menarche, gender, weight, height, nationality, current residency, marital status, highest educational level, occupational status, household income (HHI), and comorbidities. These questions aimed to provide a comprehensive overview of the participants’ backgrounds and to identify any sociodemographic factors that might influence their diagnostic experiences and satisfaction levels.
2. PCOS Diagnosis Experience and Satisfaction:
  ○ This section focused on assessing the participants’ experiences with the PCOS diagnosis process and their satisfaction with the information and support they received. It included 14 questions adapted from a previously validated questionnaire. The questions covered various aspects of the diagnostic process, including the following:
    ▪ Time from symptom onset to seeking medical attention.
    ▪ Time from seeking medical attention to receiving a PCOS diagnosis.
    ▪ Number of visits to health professionals before diagnosis.
    ▪ Manner in which the diagnosis was conveyed.
    ▪ Adequacy of the information provided about PCOS, its management, and potential complications.
    ▪ Emotional support and counseling received after diagnosis.
    ▪ Overall satisfaction with the diagnostic experience.

### 2.4. Translation and Validation

To ensure the questionnaire was culturally relevant and comprehensible for the target population, it was translated from English into Arabic by a group of four native Arabic speakers. The translation process included forward and backward translations to maintain the accuracy and integrity of the questions. The translated questionnaire was then reviewed by a panel of experts in the field to ensure its validity and reliability.

A pre-test of the questionnaire was conducted with a small sample of women diagnosed with PCOS to assess its clarity and relevance. Feedback from the pre-test was used to refine and adjust the questionnaire, improving its overall quality and ensuring it effectively captured the intended data.

### 2.5. Data Collection Procedure

Participants were recruited through various online and offline channels to ensure a broad and diverse sample. Recruitment efforts included social media platforms such as Facebook, Twitter, and Instagram, where the survey link was shared in groups and communities focused on women’s health and PCOS support. Additionally, the link was disseminated in online forums and support groups dedicated to PCOS. Medical institutions and clinics played a crucial role in the recruitment process as well. Collaborations with gynecologists and endocrinologists in major hospitals and clinics across Saudi Arabia facilitated the distribution of flyers and posters containing the survey link and QR code in waiting areas. Furthermore, email invitations were sent to contacts within medical institutions, requesting their assistance in distributing the survey to eligible patients.

The survey was administered online using Google Forms, selected for its user-friendly interface, accessibility, and secure data collection capabilities. Upon accessing the survey link, participants were presented with an informed consent form that outlined the study’s purpose, procedures, potential risks and benefits, confidentiality measures, and the voluntary nature of participation. Participants had to agree to the terms of the informed consent form before proceeding to the survey questions. The survey comprised two main sections: sociodemographic data and PCOS diagnosis experience and satisfaction. The sociodemographic section included questions on age, age at menarche, weight, height, nationality, residency, marital status, educational level, occupational status, household income, and comorbidities. The PCOS diagnosis experience and satisfaction section assessed the time from symptom onset to seeking medical attention, the time from seeking medical attention to receiving a diagnosis, the number of visits to health professionals before diagnosis, how the diagnosis was conveyed, the adequacy of information provided, emotional support received, and overall satisfaction with the diagnostic process.

To ensure comprehensive data collection, participants were encouraged to complete all questions, with mandatory fields included to prevent incomplete responses. The estimated time to complete the survey was 10–15 min. Quality control measures were implemented to enhance the reliability and validity of the data. The questionnaire was pre-tested with a small sample of women diagnosed with PCOS to identify any issues with clarity or relevance, and feedback was used to make necessary adjustments before the full launch. Data collection was regularly monitored to ensure the survey was functioning correctly and to address any technical issues promptly. Participants’ responses were anonymized, and data were stored securely, with only the research team having access to the raw data for analysis.

### 2.6. Ethical Considerations

This study received ethical approval from the Institutional Review Board (IRB) of King Faisal University (IRB log number: ETHICS1865). Participants provided informed consent after being briefed on the study’s purpose, procedures, risks, benefits, and confidentiality measures. Participation was voluntary, and anonymity was maintained by not collecting personal identifiers. Data were securely stored and accessed only by the research team. The questionnaire was designed to avoid distress, focusing solely on diagnostic and management experiences of PCOS. These ethical practices ensured participant privacy and data protection.

### 2.7. Statistical Analysis

Data were analyzed using IBM SPSS software version 29. Descriptive statistics summarized demographic characteristics, presenting frequencies and percentages for categorical variables and means with standard deviations for continuous variables. Inferential statistics, including linear regression analysis, identified predictors of satisfaction with the PCOS diagnosis process, considering factors such as age, BMI, marital status, and time since diagnosis. Statistical significance was set at a *p*-value of 0.05. The analysis also examined the impact of diagnostic timelines and regional variations on satisfaction levels. These analyses aimed to uncover factors influencing diagnostic experiences and identify areas for improving patient care.

## 3. Results

The study sample (Table 1) predominantly comprised women aged 21–40 years (82.3%), with a mean age of 28.6 years, indicating that the majority of participants were in their reproductive years. The BMI distribution showed that 40.3% of women had a normal BMI, while a significant portion was overweight (28.1%) or obese (26.4% across three obesity classes), highlighting a common comorbidity in PCOS. The majority of participants were Saudi nationals (88.7%), reflecting the study’s geographic focus. Nearly half of the participants were single (49.2%), with married women comprising 45.9%.

Educationally, the sample was highly educated, with 71.2% holding bachelor’s degrees and 10.2% having post-graduate qualifications. Regionally, the central (40.8%) and western (36.1%) areas were well represented. In terms of occupation, the participants were diverse, including employees (26.8%), housewives (17.7%), and students (29.3%). A considerable proportion (50.4%) reported a monthly income above SAR 10,000, indicating a relatively high socioeconomic status.

The mean age at menarche was 12.7 years, with most experiencing menarche between 11 and 15 years. The average age at PCOS diagnosis was 23.1 years, and the time from symptom onset to seeking medical attention varied, with 43.2% seeking help within a year. Diagnosis typically occurred within 1–5 months of seeking medical attention (64.2%). Most participants had been diagnosed for three or more years (55.9%).

Regarding the diagnostic process, 59.6% of participants required 1–2 visits to health professionals before diagnosis. The vast majority were informed of their diagnosis during a doctor’s visit (96.4%). However, information adequacy was a concern, with only 42.7% considering the information provided at diagnosis to be adequate, while 41.6% found it inadequate, and 15.7% received no information.

Table 2 illustrates the specific needs and challenges faced by patients with PCOS during the diagnostic process and their satisfaction with healthcare providers. The highest satisfaction was reported for information given about PCOS, with 42.6% of participants being very satisfied and only 7.1% receiving no information. Satisfaction with information on lifestyle management and medical therapy was slightly lower, with 39.5% and 41.8% being very satisfied, respectively. However, a significant portion of patients were dissatisfied or received no information about long-term complications, with only 32.9% being very satisfied and 22.2% receiving no information. Emotional support and counseling after diagnosis were also areas of concern, with 30.1% being very dissatisfied and 39.2% being very satisfied. Information regarding potential infertility showed the lowest satisfaction, with 33.8% being very dissatisfied and only 29.9% being very satisfied.

Table 3 presents the results of a linear regression analysis examining the factors influencing satisfaction with the PCOS diagnosis experience and the effect of time since diagnosis. The model includes various predictors, with the constant being non-significant (*p* = 0.302). Significant positive predictors of satisfaction include age of participants (B = 0.174, *p* = 0.001), indicating that older women tend to be more satisfied with their diagnosis experience, and height (B = 26.035, *p* = 0.016), suggesting that taller women report higher satisfaction. Conversely, marital status (B = −1.137, *p* = 0.024) and time since diagnosis (B = −0.872, *p* = 0.012) are significant negative predictors, indicating that married women and those with a longer time since diagnosis tend to be less satisfied. Other variables such as age at menarche, weight, BMI, nationality, educational level, monthly income, and age at diagnosis did not show significant effects on satisfaction.

Table 4 presents the results of a linear regression model analyzing the effect of different timelines on the satisfaction of women with their PCOS diagnosis experience. The constant value (B = 3.222, *p* = 0.005) provides the baseline satisfaction level. The time from the onset of symptoms until seeking medical attention shows a significant negative impact on satisfaction (B = −0.631, *p* = 0.026), indicating that longer delays in seeking medical attention are associated with lower satisfaction. Conversely, the time from seeking medical attention to receiving a PCOS diagnosis positively impacts satisfaction (B = 1.384, *p* = 0.000), suggesting that quicker diagnoses lead to higher satisfaction levels. Additionally, the number of visits to health professionals before diagnosis positively influences satisfaction (B = 1.181, *p* = 0.000), implying that more consultations before diagnosis contribute to greater satisfaction. Lastly, being satisfied with how the PCOS diagnosis was conveyed significantly enhances overall satisfaction (B = 3.380, *p* = 0.000).

## 4. Discussion

This study provides valuable insights into the diagnostic experiences of women with Polycystic Ovary Syndrome (PCOS) in Saudi Arabia, highlighting several key areas that warrant discussion. The findings reveal important aspects of the diagnostic process, patient satisfaction, and areas for potential improvement in healthcare delivery for women with PCOS.

### 4.1. Diagnostic Timeline and Process

The study results indicate that a significant proportion of women (43.2%) sought medical attention within a year of symptom onset. This relatively prompt health-seeking behavior is encouraging and may reflect increasing awareness of PCOS symptoms among Saudi women. However, it is concerning that 42.1% waited 1–4 years, and 11.8% waited 5 or more years before seeking medical attention. This delay in seeking care is consistent with findings from other studies, which have shown that women with PCOS often experience significant delays in diagnosis [29]. The reasons for these delays may include lack of awareness about PCOS symptoms, normalization of irregular menstrual cycles, or hesitancy to discuss reproductive health issues due to cultural factors [30].

Once medical attention was sought, the majority of women (64.2%) received a diagnosis within 1–5 months. This relatively quick diagnostic timeline is positive and suggests that healthcare providers in Saudi Arabia are becoming more adept at recognizing and diagnosing PCOS. However, it is important to note that 28.6% of women experienced a delay of 6 months or more in receiving a diagnosis after seeking medical attention. This highlights the need for continued education and training for healthcare providers to improve early recognition and diagnosis of PCOS [31].

The finding that 59.6% of women required only one to two visits to health professionals before receiving a diagnosis is encouraging. However, 40.4% required three or more visits, which may indicate challenges in the diagnostic process or the need for multiple specialist consultations. This aligns with previous research showing that women with PCOS often consult multiple healthcare providers before receiving a definitive diagnosis [32,33].

### 4.2. Information Provision and Patient Satisfaction

A critical finding of this study is the inadequacy of information provided to patients at the time of diagnosis. Only 42.7% of women considered the information they received about PCOS at diagnosis to be adequate, while 41.6% found it inadequate, and 15.7% received no information at all. This lack of comprehensive information at diagnosis is a significant concern, as it may lead to poor understanding of the condition, its management, and potential long-term health implications [34,35].

The satisfaction levels regarding different aspects of information provision varied considerably. While information about PCOS itself and medical therapy was relatively well received (42.6% and 41.8% of women were very satisfied, respectively), there were notable gaps in other areas. Information about long-term complications and potential infertility showed particularly low satisfaction rates, with 22.2% and 33.8% of women receiving no information on these topics, respectively. This finding aligns with previous studies that have highlighted the need for more comprehensive patient education in PCOS management [36].

The low satisfaction with emotional support and counseling after diagnosis (30.1% women were very dissatisfied) is particularly concerning. PCOS can have significant psychological impacts, including increased risks of anxiety and depression [37,38]. The lack of adequate emotional support at the time of diagnosis represents a missed opportunity for early intervention and support that could potentially mitigate these psychological risks [39].

Dissatisfaction with the diagnostic process and the information provided by healthcare professionals for PCOS is a widespread issue. Many women globally, including those in Saudi Arabia, experience delays, misdiagnosis, and inadequate explanations about their condition [40]. In this study, only 42.7% of participants found the information at diagnosis to be adequate, with many receiving insufficient or no information. This lack of comprehensive education exacerbates the challenges of managing PCOS, particularly regarding its long-term implications like infertility and metabolic risks. Addressing these gaps through standardized protocols and better training for healthcare providers is essential to improve patient satisfaction and outcomes in managing PCOS [40].

### 4.3. Factors Influencing Patient Satisfaction

The regression analysis revealed several factors influencing patient satisfaction with the PCOS diagnosis experience. The positive association between age and satisfaction suggests that older women may have more realistic expectations or better coping mechanisms when dealing with chronic health conditions [41,42]. The negative association between marital status and satisfaction, with married women reporting lower satisfaction, could reflect the added stress of fertility concerns or the impact of PCOS on marital relationships [43].

Interestingly, the time since diagnosis was negatively associated with satisfaction, indicating that women who have lived with the diagnosis longer tend to be less satisfied with their initial diagnostic experience [44,45]. This could be due to retrospective dissatisfaction as women learn more about their condition over time or experience ongoing challenges in managing PCOS [46].

The positive impact of quicker diagnoses and more healthcare visits before diagnosis on satisfaction levels underscores the importance of thorough evaluation and timely diagnosis in PCOS management. This finding supports the need for comprehensive diagnostic protocols and patient-centered care approaches in PCOS [47].

### 4.4. Infertility and PCOS

Infertility is a significant concern for women with Polycystic Ovary Syndrome (PCOS), as the hormonal imbalances and anovulation associated with the condition can greatly reduce fertility potential [48]. PCOS is one of the leading causes of infertility in women of reproductive age, often requiring medical intervention for conception. The management of infertility in PCOS is complex and typically involves lifestyle modifications, pharmacological treatments, and sometimes assisted reproductive technologies (ART) [49]. Beyond the immediate challenge of achieving pregnancy, infertility in PCOS also has profound psychological impacts, contributing to increased stress, anxiety, and depression among affected women [50].

The 2024 European Society of Hypertension (ESH) statement on hypertensive disorders of pregnancy underscores the importance of addressing infertility in women with PCOS, particularly given the increased risks associated with pregnancy complications such as hypertensive disorders. This statement highlights the need for careful preconception counseling and management to optimize outcomes for both the mother and the fetus in women with PCOS that are pregnant [51]. Integrating these considerations into the broader management of PCOS is crucial for improving the long-term reproductive health and overall well-being of affected women.

### 4.5. Regional and Healthcare System Implications

The study’s findings have important implications for healthcare delivery in Saudi Arabia. The predominance of participants from central and western regions (76.9% combined) suggests potential regional disparities in PCOS awareness or access to specialized care. This regional imbalance highlights the need for more equitable distribution of PCOS-related healthcare resources and education across the country [52].

The high educational level of the sample (81.4% women with bachelor’s degree or higher) and the relatively high socioeconomic status (50.4% women with monthly income above SAR 10,000) may not be representative of the broader Saudi population. This potential sampling bias should be considered when interpreting the results and may indicate disparities in access to PCOS diagnosis and care based on educational and socioeconomic factors [53,54,55].

### 4.6. Limitations and Future Research Directions

This study has several limitations that should be acknowledged. The online survey methodology may have introduced selection bias, potentially over-representing women with higher education and socioeconomic status. Future studies could employ more diverse recruitment strategies to ensure a more representative sample.

The cross-sectional nature of the study limits the ability to draw causal inferences. Longitudinal studies tracking women from symptom onset to diagnosis and management could provide more comprehensive insights into the journey of patients with PCOS.

The study focused primarily on the diagnostic experience and did not explore in-depth the long-term management experiences of women with PCOS. Future research could investigate the ongoing challenges and needs of women living with PCOS in Saudi Arabia, including access to specialized care, long-term health outcomes, and quality of life impacts.

Additionally, qualitative research exploring the perspectives of both patients and healthcare providers could offer valuable insights into the barriers and facilitators of effective PCOS diagnosis and management in the Saudi healthcare system.

## 5. Conclusions

This retrospective study of PCOS diagnostic experiences among Saudi women has shed light on critical aspects of healthcare delivery and patient satisfaction in Saudi Arabia. The findings reveal a complex landscape where prompt health-seeking behavior coexists with significant delays in diagnosis for many women. The study underscores the urgent need for improvements in several key areas: comprehensive patient education, particularly regarding long-term complications and fertility implications; enhanced emotional support and counseling; and standardized diagnostic protocols across healthcare settings. The variability in patient satisfaction, influenced by factors such as age, marital status, and time since diagnosis, highlights the importance of personalized, patient-centered care approaches. Regional disparities in PCOS awareness and care access point to the necessity for a more equitable distribution of healthcare resources and targeted public health campaigns. While the study demonstrates some positive aspects of PCOS care in Saudi Arabia, such as relatively quick diagnoses for many patients, it also reveals substantial room for improvement in information provision and emotional support. These findings have significant implications for clinical practice, healthcare policy, and future research directions. By addressing the identified gaps and leveraging the insights gained from this study, healthcare providers and policymakers have the opportunity to substantially enhance the quality of care for women with PCOS in Saudi Arabia. This research not only contributes valuable knowledge to the field of women’s health in the region but also provides a foundation for developing targeted interventions and policies aimed at improving PCOS diagnosis, management, and overall patient outcomes. Moving forward, it is crucial to implement these findings into practice, continue research efforts to address remaining questions, and regularly evaluate the effectiveness of any implemented changes to ensure ongoing improvement in PCOS care across Saudi Arabia.

## Figures and Tables

**Table 1:** Sociodemographic characteristics of the studied sample (n = 1182).

		Frequency	Percent
Age	<20 years	105	8.9
	21–40 years	973	82.3
	41–60 years	97	8.2
	>60 years	7	0.6
	Mean (SD)	28.6 (8.0)	
	Range	18–66 years	
BMI category	Underweight	61	5.2
	Normal	476	40.3
	Overweight	332	28.1
	Obese Class 1	205	17.3
	Obese Class 2	69	5.8
	Obese Class 3	39	3.3
	Mean (SD)	26.7 (6.4)	
	Range	14.0–69.6	
Nationality	Non-Saudi	134	11.3
	Saudi	1048	88.7
Marital status	Single	581	49.2
	Married	543	45.9
	Divorced/widowed	58	4.9
Education level	School	221	18.7
	Bachelors	841	71.2
	Post-graduate	120	10.2
Region	Central	482	40.8
	Western	427	36.1
	Eastern	134	11.3
	North	81	6.9
	South	58	4.9
Occupation	Unemployed	205	17.3
	Employee	317	26.8
	Housewife	209	17.7
	Non-medical students	188	15.9
	Medical students	158	13.4
	Medical personnel	92	7.8
	Retired	13	1.1
Monthly income	SAR <5000	177	15.0
	SAR 5000–10,000	409	34.6
	SAR >10,000	596	50.4
Age of menarche	<11 years	101	8.5
	11–15 years	999	84.5
	>15 years	82	6.9
	Mean (SD)	12.7 (1.8)	
	Range	7–24	
Age at diagnosis of PCOS	Mean (SD)	23.1 (6.2)	
	Range	11–49	
Time from onset of symptoms until seeking medical attention	<1 year	511	43.2
	1–4 years	498	42.1
	5 or more years	140	11.8
	I don’t remember	33	2.8
Time from seeking medical attention to a diagnosis of PCOS	<1 month	68	5.8
	1–5 months	759	64.2
	>6 months	338	28.6
	I don’t remember	16	1.4
Time since diagnosis (in years)	<1 year	258	21.8
	1–2 years	263	22.3
	≥3	661	55.9
Number of visits to professionals before diagnosis	1–2	704	59.6
	3–4	342	28.9
	≥5	136	11.5
Information about PCOS diagnosis			
How were you informed of diagnosis	By telephone	43	3.6
	During doctor’s visit	1139	96.4
Given/referred to information at PCOS diagnosis	No	185	15.7
	Yes (inadequate)	492	41.6
	Yes (adequate)	505	42.7

Obese Class 1: BMI 30–34.9; Obese Class 2: BMI 35–39.9; and Obese Class 3: BMI ≥ 40.

**Table 2:** Specific needs and challenges faced during PCOS diagnosis by patients and their satisfaction regarding their healthcare provider.

		No Information Provided	Very Dissatisfied	Neither	Very Satisfied
Satisfaction with information given about PCOS	N	84	270	325	503
	%	7.1	22.8	27.5	42.6
Satisfaction with information given about lifestyle management	N	145	284	286	467
	%	12.3	24.0	24.2	39.5
Satisfaction with information given about medical therapy	N	82	309	297	494
	%	6.9	26.1	25.1	41.8
Satisfaction with information about long-term complications	N	262	302	229	389
	%	22.2	25.5	19.4	32.9
Satisfaction with healthcare provider emotional support and counseling after diagnosis	N	0	356	363	463
	%	0.0	30.1	30.7	39.2
Satisfaction with information given about potential infertility	N	399	233	197	353
	%	33.8	19.7	16.7	29.9

Legend: Obese Class 1 (BMI 30–34.9), Obese Class 2 (BMI 35–39.9), and Obese Class 3 (BMI ≥ 40) are based on the participants’ current Body Mass Index (BMI) at the time of the survey.

**Table 3:** Linear regression of factors and effect of timeline since PCOS diagnosis on the satisfaction.

	Unstandardized Coefficients		Standardized Coefficients	Sig.	95% CI	
	B	SE	Beta		Lower Bound	Upper Bound
(Constant)	−17.462	16.908		0.302	−50.635	15.710
Age of participants (years)	0.174	0.053	0.166	0.001 *	0.070	0.279
Age of menarche (years)	−0.048	0.133	−0.010	0.721	−0.309	0.214
Weight (kg)	−0.194	0.100	−0.377	0.053	−0.391	0.003
Height (m)	26.035	10.754	0.225	0.016 *	4.936	47.133
BMI	0.444	0.245	0.342	0.071	−0.038	0.925
Nationality (Saudi)	−0.439	0.798	−0.017	0.582	−2.006	1.127
Marital status (married)	−1.137	0.502	−0.080	0.024 *	−2.122	−0.152
Educational level	−0.887	0.475	−0.056	0.062	−1.818	0.044
Monthly income	0.392	0.363	0.034	0.281	−0.321	1.105
Age at diagnosis	−0.078	0.063	−0.058	0.216	−0.201	0.045
Time since diagnosis (years)	−0.872	0.348	−0.085	0.012 *	−1.555	−0.190

* significance of difference.

**Table 4:** Effect of different timelines on the satisfaction of women regarding PCOS diagnosis experience (linear regression model).

	Unstandardized Coefficients		Standardized Coefficients	Sig.	95% CI for B	
	B	SE	Beta		Lower Bound	Upper Bound
(Constant)	3.222	1.155		0.005	0.956	5.487
Time from onset of symptoms until seeking medical attention	−0.631	0.283	−0.054	0.026	−1.186	−0.075
Time from seeking medical attention to receiving a PCOS diagnosis	1.384	0.394	0.090	0.000	0.612	2.157
Number of visits to professionals before diagnosis	1.181	0.311	0.097	0.000	0.571	1.792
Satisfied with manner in which you were informed of PCOS diagnosis (yes)	3.380	0.141	0.576	0.000	3.103	3.657

## Data Availability

Data are available upon request.
